# 381. The Importance of Data Accuracy and Transparency for Policymaking During a Public Health Crisis: A Case Study in the State of Iowa

**DOI:** 10.1093/ofid/ofab466.582

**Published:** 2021-12-04

**Authors:** Megan L Srinivas, HyungSub Shim, Dana Jones, Patrick R Hansen, Sara A Willette, Auriel Willette, E Rosalie Li-Rodenborn, Eli N Perencevich, Michihiko Goto

**Affiliations:** 1 University of North Carolina, Ames, Iowa; 2 University of Iowa Carver College of Medicine, Iowa City, Iowa; 3 University of Iowa Hospitals and Clinics, Iowa City, Iowa; 4 novéInsights, Grinnell, Iowa; 5 Iowa COVID-^19^ Tracker, Ames, Iowa; 6 Iowa State University, Ames, Iowa; 7 Johns Hopkins Bloomberg School of Public Health, Baltimore, Maryland; 8 University of Iowa, Iowa City, Iowa

## Abstract

**Background:**

High-quality data are necessary for decision-making during the SARS-CoV-2 pandemic. Lack of transparency and accuracy in data reporting can erode public confidence, mislead policymakers, and endanger safety. Two major data errors in Iowa impacted critical state- and county-level decision-making.

**Methods:**

The Iowa Department of Public Health (IDPH) publishes daily COVID-19 data. Authors independently tracked daily data from IDPH and other publicly available sources (i.e., county health departments, news media, and social networks). Data include: number and type of tests, results, hospitalizations, intensive care unit admissions, and deaths at state/county levels.

**Results:**

Discrepancies were identified between IDPH and non-IDPH data, with at least two confirmed by IDPH: (1) The backdating of test results identified on May 28, 2020. IDPH labeled results as occurring up to four months before the actual test date. IDPH confirmed that if a person previously tested for SARS-CoV-2, a new test result was attributed to the initial test’s date. Corrections on August 19, 2020 increased positivity rates in 31 counties, but decreased the state’s overall rate (9.1% to 7.5%). (2) The selective exclusion of antigen test results noted on August 20, 2020. Antigen testing was included in the total number of tests reported in metric denominators, but their results were being excluded from their respective numerators. Thus, positive antigen results were interpreted as de facto negative tests, artificially lowering positivity rates. Corrections increased Iowa’s positivity rate (5.0% to 14.2%). In July 2020, the Iowa Department of Education mandated in-person K-12 learning for counties with < 15% positivity. These data changes occurred during critical decision-making, altering return-to-learn plans in seven counties. The Center for Medicare and Medicaid Services’ requirements also caused nursing homes to urgently revise testing strategies.

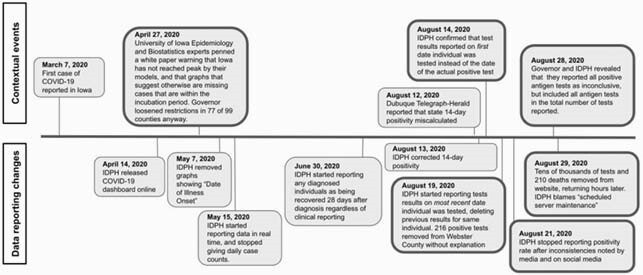

Timeline of changes to Iowa state COVID-19 testing through the end of August 2020.

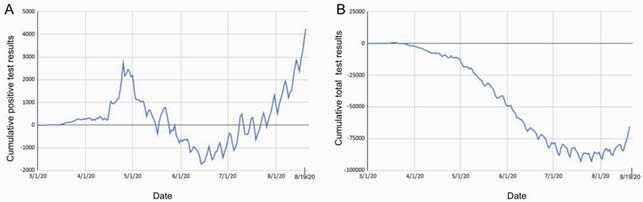

Change in positive and overall test results due to IDPH data corrections. These graphs represent the difference in cumulative total reported test results when pulled from the IDPH website on September 29, 2020 compared to data for the same dates when pulled on August 19, 2020 before the announced adjustment. The adjustment and subsequent daily changes in reported data amount to a dramatic change in the number of reported positive cases (A) with an increase of nearly 3,000 cases by April 25, as well as the loss of tens of thousands of data points when tracking total resulted tests (B).

**Conclusion:**

Data availability, quality, and transparency vary widely across the US, hindering science-based policymaking. Independent audit and curations of data can contribute to better public health policies. We urge all states to increase the availability and transparency of public health data.

**Disclosures:**

**All Authors**: No reported disclosures

